# Refined genetic maps reveal sexual dimorphism in human meiotic recombination at multiple scales

**DOI:** 10.1038/ncomms14994

**Published:** 2017-04-25

**Authors:** Claude Bhérer, Christopher L. Campbell, Adam Auton

**Affiliations:** 1 Department of Genetics, Albert Einstein College of Medicine, 1301 Morris Park Avenue, Bronx, New York 10461, USA

## Abstract

In humans, males have lower recombination rates than females over the majority of the genome, but the opposite is usually true near the telomeres. These broad-scale differences have been known for decades, yet little is known about differences at the fine scale. By combining data sets, we have collected recombination events from over 100,000 meioses and have constructed sex-specific genetic maps at a previously unachievable resolution. Here we show that, although a substantial fraction of the genome shows some degree of sexually dimorphic recombination, the vast majority of hotspots are shared between the sexes, with only a small number of putative sex-specific hotspots. Wavelet analysis indicates that most of the differences can be attributed to the fine scale, and that variation in rate between the sexes can mostly be explained by differences in hotspot magnitude, rather than location. Nonetheless, known recombination-associated genomic features, such as THE1B repeat elements, show systematic differences between the sexes.

## Abstract (web summary)

It is known that males have lower recombination rates than females over much of the genome but little is known about differences at a fine scale. Here the authors combine data from over 100,000 meioses and show that the majority of differences can be explained by variation in hotspot magnitude.

In humans and other mammals, meiosis in females and males differs both in terms of timing and in the number of gametes produced. As an essential outcome of meiosis, recombination also differs substantially between the sexes. Genome wide, human female crossover rates are ∼1.6-fold greater than that of males^1^,^2^,^3^,^4^,^5^. Sexual dimorphism in human recombination is not limited to simple changes to the overall recombination rate, but also includes differences in the distribution of recombination throughout the genome. Specifically, male autosomal recombination rates tend to be lower than female rates over the majority of the genome, but tend to be elevated towards to the telomeres^1^,^2^,^5^. While these broad-scale differences have been known for decades, the fine-scale differences in recombination between the sexes remain to be fully characterized.

Nonetheless, previous large-scales studies of recombination have characterized differences between females and males^2^,^4^, and identified a number of variants associated with sex-specific recombination rates^2^,^6^,^7^,^8^,^9^,^10^. These findings imply that the genetic regulation of meiotic recombination has evolved to be different in females and males. Yet, how recombination rates are regulated along chromosomes in a sex-specific manner is largely unknown.

In this paper, we build new sex-specific genetic maps based on crossovers from more than 100,000 meioses obtained from published pedigree studies. These maps reveal the variation of recombination rates in females and males throughout the genome at an unprecedented resolution. We characterize this variation over a range of scales using wavelet analysis and investigate the genomic and epigenomic features associated with the sex differences. We show that female and male differences in recombination rate can be mainly attributable to the fine scale, and find sexually dimorphic patterns of recombination in THE1B elements and gene promoter regions.

## Results

### Building refined sex-specific genetic maps

To study recombination differences between the sexes, we assembled a collection of recombination events from six recent studies of human pedigrees^2^,^6^,^7^,^11^,^12^,^13^,^14^, pertaining to a total of 104,246 informative meioses (57,919 female and 46,327 male meioses) (Table 1). The vast majority of meioses are derived from individuals of European ancestry, representing 93.7% of all meioses, and 6.3% are from other origins including African American (1.6%), East Asian (1.8%) and Latino American (1.5%) (Supplementary Table 1). The combined data set consists of 2,338,628 female and 999,007 male recombination events (Table 1, Supplementary Tables 2 and 3). The boundaries of these recombination events define a total of 833,754 and 18,039 single nucleotide polymorphism (SNPs) intervals on the autosomal and X chromosomes, respectively.

**Figure 1:**
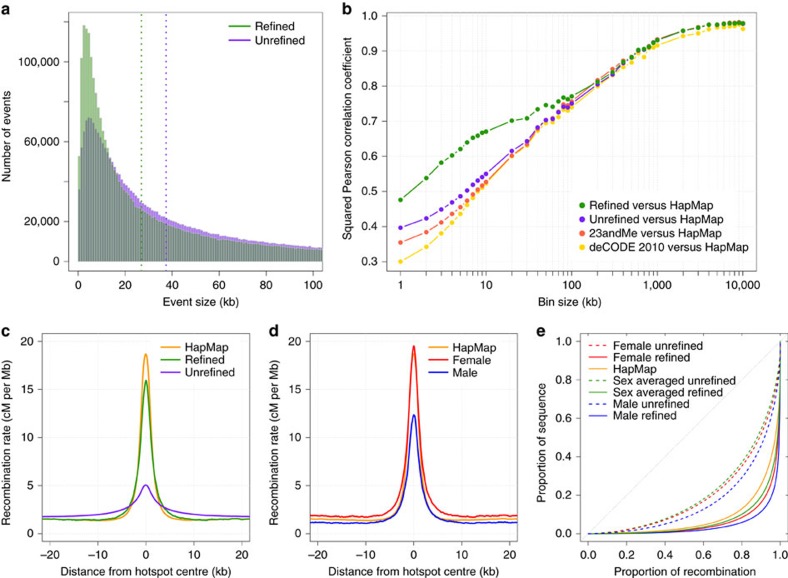
Improved resolution of the refined genetic maps. (**a**) Localization of recombination events before and after refinement by the MCMC procedure. (**b**) Squared Pearson correlation as a function of scale between the sex-averaged maps and the HapMap map. (**c**) Mean recombination rate around hotspots defined in the LD-based HapMap map before and after refinement. (**d**) Sex-specific recombination rates in the refined map around HapMap recombination hotspots. (**e**) Cumulative proportion of recombination in the genetic map versus the proportion of sequence.

We constructed high-resolution female and male maps, by modelling the recombination rate in each inter-SNP interval using a Bayesian Markov Chain Monte Carlo (MCMC) procedure based on the assumption that the fine-scale recombination rate is similar across individuals of a given sex (Methods and Supplementary Methods). By combining information across multiple families, this method allows the refinement and localization of recombination events, and therefore allows recombination rates to be estimated at a finer scale. Applying this method to female and male separately, we obtained female and male mean posterior rates for each inter-SNP interval as well as credible intervals (CI). In addition to the resulting ‘refined' sex-specific genetic maps, we generated a sex-averaged refined map by averaging female and male rates in each interval. After refinement, the median resolution of autosomal recombination events is 26.9 kb, with 28.6% of events resolved to within 10 kb, compared to 34.7 kb resolution and 19.5% of events within 10 kb in the unrefined map (Fig. 1a). The female and male refined maps have similar median resolutions of autosomal recombination events of 27.3 kb and 26.1 kb, respectively.

To assess the accuracy of our posterior estimation of recombination rates, we compared the refined maps to maps generated using standard methods. The female and male genetic maps constructed with our method are in good agreement with previous pedigree-based maps^2^,^4^ (Supplementary Fig. 1), although it should be noted that we have reused the data use to generate these maps. A cleaner comparison can be made with the HapMap map^15^ generated from patterns of linkage disequilibrium (LD), where our sex-averaged refined map shows a Pearson *r*^*2*^>0.93 at the megabase scale (Fig. 1b). At the fine scale, where LD-based maps are expected to have higher resolution, the sex-averaged refined map shows an increased correlation to the HapMap map compared to the unrefined map and to the earlier pedigree-based maps. For example, at the 5 kb scale, the Pearson correlation between our map and the HapMap map is *r*^*2*^=0.62, whereas for the unrefined map it is 0.49, and respectively 0.46 and 0.44 for the Campbell *et al*.^2^ map and the deCODE 2010 map^4^. Similarly, the refined map shows improved correlation at scales below 200 kb to the LD-based CEU map generated from 1,000 Genomes Project Illumina OMNI array data^16^ (Supplementary Fig. 2b). Refinement also improves correlation for a refined Icelandic map constructed using data from Kong *et al*.^7^ only (Supplementary Fig. 3).

To evaluate the potential impact of the different ancestries in the refined map, we build a refined European map applying our method to the recombination events inferred in parents of European ancestry, representing 94.3% of female and 93% of male meioses included in the combined data set (Supplementary Table 1). The refined female, male and sex-averaged maps are highly correlated to the European maps at all scales (Supplementary Fig. 4). At the 20 kb scale, Pearson's *r*^*2*^ between the refined and European maps is above 0.96. At the 1 Mb scale and beyond, they have near perfect correlation. The two maps also have the same Pearson correlation to the HapMap and OMNI CEU maps suggesting that genome wide the refined estimates of recombination rates are mostly representative of Europeans.

We next tested the ability of our method to narrow down the location of recombination events to known recombination hotspots inferred from the HapMap data^17^. In the refined map, the mean recombination rate around hotspot centres is much higher than for the unrefined map, and is much more comparable to the rates estimated by HapMap (Fig. 1c). Likewise, the refined map shows marked improvement in the localization of events compared to previous pedigree maps (Supplementary Fig. 2c). Increased recombination rates are observed at LD-inferred hotspots in both males and females (Fig. 1d). While the elevation in recombination rate observed at hotspots is higher in females, the fold increase over the background rate is similar between the sexes (females: 19.5 cM per Mb/2.0 cM per Mb=9.75. Males: 12.3 cM per Mb/1.3 cM per Mb=9.46).

Compared to the unrefined map, the refined map shows a higher proportion of recombination concentrated in a smaller fraction of total sequence (Fig. 1e). In the sex-averaged refined map, 80% of recombination is localized in 10.6% of the genome, which is comparable to 13.5% in the HapMap, and much more concentrated than the 38.5% observed for the unrefined map. This is consistent with a better localization of events to hotspots of recombination in the refined map. Male recombination occurs in a narrower portion of the genome than female, with 80% of male recombination occurring in 6.1% of sequence compared to 8.8% for female. This is consistent with male recombination being slightly more concentrated in hotspots^2^.

### The fine-scale sexual dimorphism in recombination landscape

**Figure 2:**
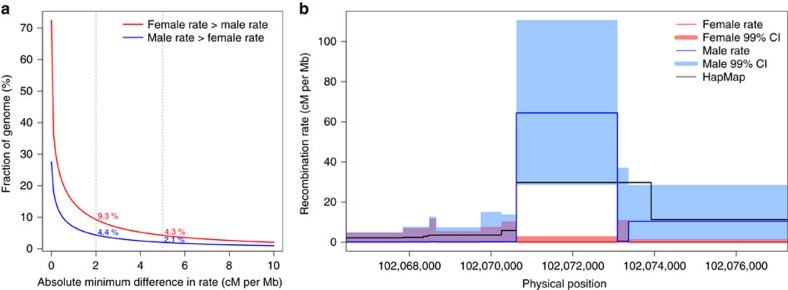
Fine-scale differences in recombination rates between females and males. (**a**) Fraction of the autosomal genome with female recombination rate higher than the male rate (red) and male rate higher than female (blue). (**b**) An example of sex-specific hotspot on chromosome 15, recombining at higher rate in males (blue) than females (red). The 10 kb dimorphic region is centred around an inter-SNP interval showing significant sex-difference in rate based on the 99% credible intervals (CI) computed in our MCMC mapping method.

Our sex-specific genetic maps allowed us to investigate sexual dimorphism in recombination rate at high resolution. We first sought to quantify the magnitude of differences in rate across the human genome (Fig 2a). We find 13.7% of the autosomal genome has a recombination rate difference of at least 2 cM per Mb between the sexes, with 9.3% of the genome being at least 2 cM per Mb higher in females, and 4.4% being at least 2 cM per Mb higher in males. At the more extreme end, regions showing at least 5 cM per Mb difference account for 4.3% and 2.1% of the genome in females and males, respectively.

We identified localized regions of the autosomal genome with significant sex differences in recombination rate while taking into account local uncertainties in rate estimates (Fig. 2b). Specifically, we computed CI around the mean posterior estimates of recombination rates within each SNP interval. We defined dimorphic regions as 10 kb windows around SNP intervals with significant sex difference in rate at the 99% level (Fig. 2b, Methods). Doing so, we identified 6,377 putative dimorphic regions, including 5,262 regions hotter in females and 1,115 hotter in males. By definition, these regions show significant sex differences in crossover rate, with a minimum difference of 2 cM per Mb, and a median of 9.7 cM per Mb (median rate difference for regions hotter in females: 9.2 cM per Mb and males: 12.4 cM per Mb) (Supplementary Fig. 5). We observe a higher number of regions recombining in females, which is consistent with higher genome-wide female recombination rates and male recombination being concentrated in a smaller number of hotspots. We next defined sex-specific hotspots as dimorphic regions with high recombination rate in one sex (mean rate>10 cM per Mb), but very low in the other (maximum rate across all intervals within the region<1 cM per Mb) (Fig. 2b). We find 304 female specific hotspots and 147 male specific hotspots. Applying different criteria for hotspot definition consistently yielded a small number of sex-specific hotspots relative to the tens of thousands of recombination hotspots estimated in LD maps^15^,^17^ (Supplementary Table 4). Thus, in the autosomes, only a fairly low number of genomic regions have recombination limited to one sex and suppressed in the other. Dimorphic regions, including sex-specific hotspots, appear to cluster into regions that are more recombinogenic in one sex or the other (Supplementary Fig. 6). Most obviously, male recombinogenic regions are concentrated in subtelomeric regions, with 50% located within 8 Mb of chromosome ends.

### The scale-specific variation in recombination rates

**Figure 3:**
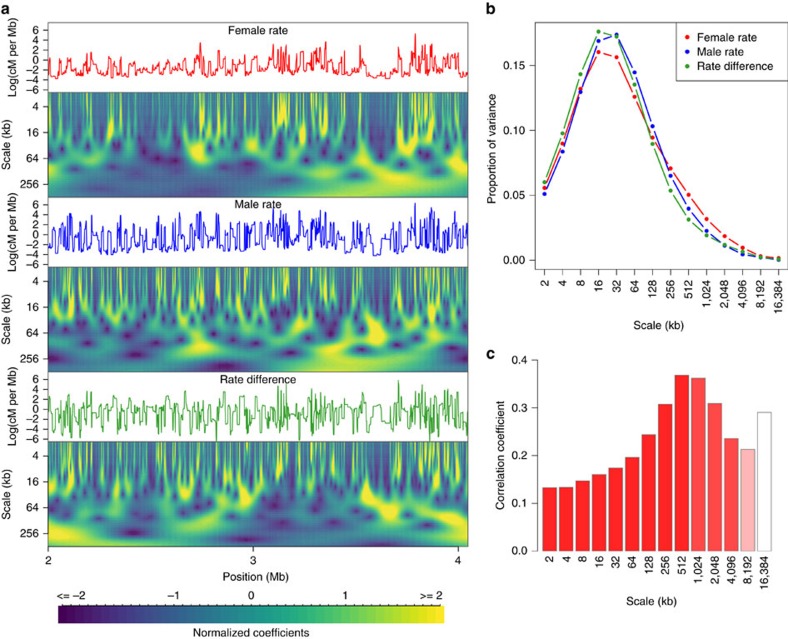
Wavelet analysis of female and male recombination rates. (**a**) Continuous wavelet transformation of recombination rates along a 2 Mb region of chromosome 10. Line plots represent the original signal to which the wavelet transform was applied, namely the female (red) and male (blue) log-transformed recombination rates, and the difference between the two (green). Scalograms represent the continuous wavelet transformation (CWT) coefficients for scales from 2 kb up to 512 kb. Colours indicate the magnitude of wavelet coefficients (blue=negative, yellow=positive) at each scale and location, with each level normalized to have equal variance. (**b**) Genome-wide power spectrum of the female and male recombination rates at scales from 2 to 16,384 kb. (**c**) Correlation between the detail coefficients of the DWT of female and male recombination rates as a function of scale. The colour of each bar indicates the *P* value of the correlation, with smaller values shown in darker red shades.

We use wavelet analysis to characterize the variation in female and male recombination rate along chromosomes at a range of scales, and assess the scale-specific differences between the sexes. Wavelet analysis allows a sequence of observations to be transformed into a series of coefficients that describe changes in the signal at each location and successively broader scales. In Fig. 3a, we illustrate wavelet transformation applied to the female recombination rate, the male rate and their difference along a 2 Mb region of chromosome 10. Sex differences in recombination rate are revealed at multiple scales, ranging from differences at specific hotspots to variation over much larger scales. This highlights an advantage of wavelet analysis, which avoids choosing an arbitrary window size at which to compare female and male recombination rates.

We performed genome-wide wavelet analysis using female and male refined maps interpolated in non-overlapping bins of 1 kb for each chromosome. More specifically, rate estimates were log_10_ transformed and the discrete wavelet transformation (DWT) was applied using the simplest wavelet Haar function for scales from 2 kb to 16 Mb (see Methods). We used the detail coefficients of the DWT to quantify the proportion of the variance in the original signal that is captured at a particular scale, and cannot be attributed to any other orthogonal scales. This measure, known as the power spectrum, is shown for female and male recombination rates, as well as their difference in Fig. 3b (Supplementary Fig. 7a). The three power spectra are very similar to each other (as are the per-chromosome power spectra—Supplementary Fig. 7b–d) and show that, genome wide, over 93% of the heterogeneity in the log recombination rate signals is captured at scales below 1 Mb (94% and 96% for male and female, respectively). Furthermore, while the fine scales (2, 4 and 8 kb) explains 28% and 26% of the variance in the male and female rate signals, the greatest contribution comes from intermediate scales (16, 32 and 64 kb), with 44% and 49% of variance explained for male and female rate, respectively.

The wavelet decomposition allows investigation of the correlation between the localized changes in the sex-specific recombination rates at each scale. The variation in female recombination rate, as captured by the detail coefficients, is significantly correlated to the variation in male recombination rate from the finest scale studied, 2 kb, to the broadest, 16 Mb (Fig. 3c, Supplementary Fig. 8). The degree of correlation increases from *r*^*2*^*=*13.3% at 2 kb scale to a maximum of *r*^*2*^*=*36.8% at 512 kb scale.

As such, local variations in the recombination rate that can be attributed to broad scales are more strongly correlated between the sexes than those at the fine scale. Conversely, removing variation that can be attributed to the fine scale increases the correlation in the residual rate estimates between the sexes. Specifically, by comparing the smoothing coefficients at each scale, we see that the correlation between male and female recombination rates increases as a function of scale (Supplementary Fig. 9). As such, we conclude that the majority of the differences in recombination rate between males and females can be attributed to the fine scale.

### Sequence features associated to sex-specific recombination

**Figure 4:**
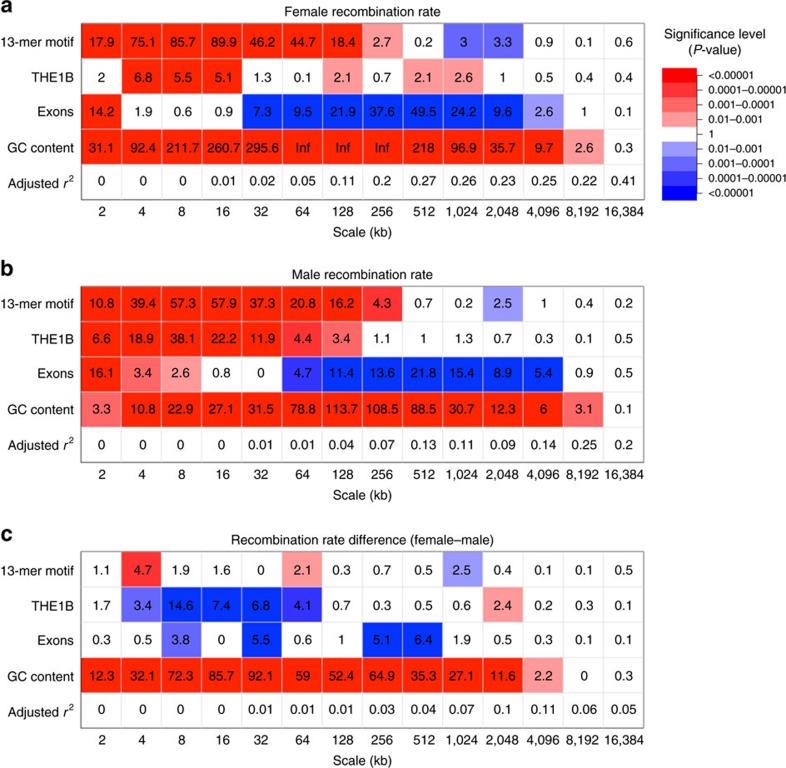
Genomic features associated with the recombination rate. Wavelet-based linear model of scale-specific genomic correlates to the recombination rate. Table shows the marginal significance (−log10 *P*-value two-sided *t*-test) for the linear model analyses of wavelet detail coefficients. Colours indicate the direction of the relationship (red=positive; blue=negative) with intensity proportional to significance. Linear regression analysis was performed on the log-transformed (**a**) female recombination rate, (**b**) male recombination rate and (**c**) on the sex difference between the log-transformed rates (female–male).

Given the availability of both of female and male recombination rate estimates, we aimed to assess the scale-specific relationship between the sex-specific rates and known recombination correlates. To do this, we performed a multiple linear regression on the wavelet coefficients at each scale using a selection of known recombination correlates as predictor variables (see Methods). In our initial analysis, we included GC content, exon density, density of THE1B repeat and density of the expected 13-base PRDM9 binding motif (CCNCCNTNNCCNC) as predictors within the model, all of which are known covariates with the sex-averaged recombination rate^5^,^15^,^18^,^19^,^20^,^21^. All of these previously associated predictors of recombination show correlations with both male and female recombination rates at some scales (Fig. 4). However, some differences in the strength of correlation emerge. For example, genome-wide GC content appears to be more strongly associated with female rate than male recombination rate over a wide range of scales (Fig. 4c, Supplementary Table 5). Conversely, THE1B elements appear to be more recombinogenic in males between scales 4 and 64 kb (Fig. 4c, Supplementary Fig. 10); a pattern that is not observed for other repeat classes (Supplementary Fig. 11). While PRDM9 motif density shows a high-positive association to both male and female recombination rate, it shows little influence on the difference between female and male rates, with the only significant (but fairly weak) correlation found at the 4 kb scale (*P*=1.8 × 10^−5^, two-sided *t*-test).

**Figure 5:**
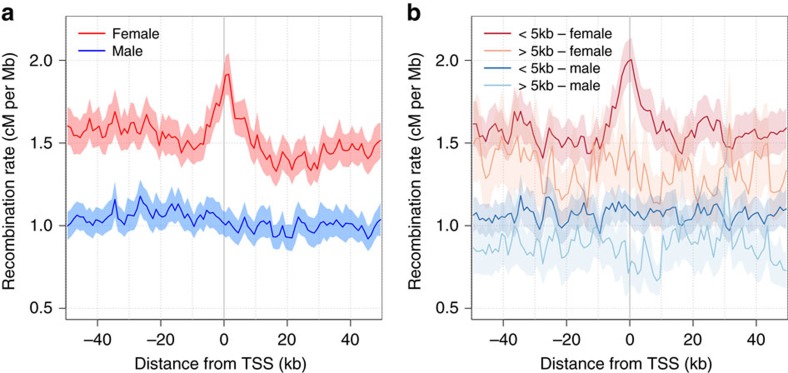
Sex-specific recombination rates around transcription start sites. Average female (red) and male (blue) recombination rates in 1 kb bins around the transcription start sites (TSS) of a subset of 15,239 GENCODE genes randomly selected to be spaced from each other by 5 kb or more. The 95% confidence interval around the mean estimates of recombination rates are shown in shaded colours. (**a**) All TSS and (**b**) TSS partitioned according to the presence/absence of a PRDM9 degenerate 13-mer motif within 5 kb.

Previous reports have shown that recombination tend to be suppressed within genes^4^,^15^,^17^,^19^. Our linear model analysis of the wavelet transformations reveals a more complex relationship between exon density and the sex-specific rates. At intermediate to large scales, exon density has a negative effect on both female and male recombination rates, whereas it has a positive effect on recombination rates at fine scales (Fig. 4a,b, Supplementary Fig. 12). To further investigate this pattern, we plotted average recombination rates around the transcription start site (TSS) of GENCODE genes. At the fine scale, we observe an elevation in female recombination rate at the TSS that is absent in males (Fig. 5a). This peak of recombination at promoter regions is consistent with previous report based on human sex-averaged rates^15^, but appears to be driven by female recombination alone. This fine-scale pattern of sexual dimorphism holds with normalized recombination rates, and both for genes located in subtelomeric and centromeric regions (Supplementary Fig. 13). The elevation of female recombination rate at the TSS appears to be associated with the co-localization of the PRDM9 motif, with a 30% rate increase from background rate being observed for genes with a 13-mer degenerate motif within 5 kb of the TSS, and no such elevation for genes without a motif within 5 kb (Fig. 5b). Furthermore, the female peak appears associated more strongly to the motif location then the TSS location itself as the recombination peak is shifted away from the TSS with increasing distance to nearest motif (Supplementary Fig. 14), which suggests a role for PRDM9 in the observed dimorphism.

Sexually dimorphic recombination in functional regions may arise as a consequence of sex differences in DNA double-strand breaks (DSBs) that initiate recombination and/or their downstream resolution. To investigate further, we used published male recombination initiation maps^22^, and find that male DSBs are significantly depleted around the TSS, with 4.4% of them overlapping a male DSB peak within 1 kb compared to an expected 5.1% (CI: 4.8–5.5%) overlap at a random position (Supplementary Fig. 15), providing independent evidence for suppressed male recombination at the promoter region of genes. Finally, we investigated recombination at CpG islands, which are known sites of transcription initiation. In females, we observe an elevation of recombination rate at these sites that is again absent in males (Supplementary Fig. 16). These different lines of evidence suggest that female meiosis is permissive of crossover activity at the site of transcription initiation whereas male meiosis suppresses recombination at these sites.

### The influence of H3K4me3 on sex-specific recombination

In females, oogenesis is initiated during early embryonic development but remains in a state of arrest until ovulation. Conversely in males, spermatogenesis occurs continuously following the first wave at puberty. As such, differences in recombination may reflect structural or epigenetic differences in the genome at the time of crossover formation. Given PRDM9's role as an H3 K4 trimethyltransferase, we investigated the association of H3K4me3 peaks with male and female recombination. Since data are currently lacking for oocytes, we focused on a testis sample^22^.

**Figure 6:**
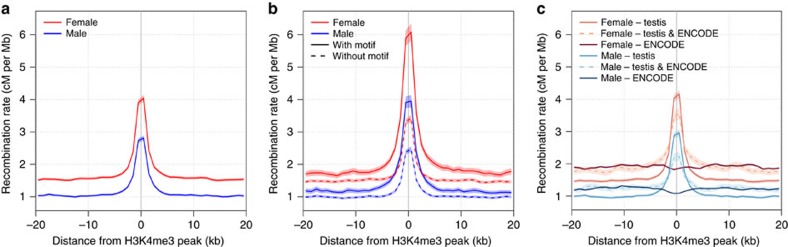
Relationship between H3K4 trimethylation and sex-specific recombination rates marks. (**a**) Average female and male recombination rates around the middle point of H3K4me3 peaks from testis sample. Recombination rates around H3K4me3 marks partitioned into (**b**) those that overlap with predicted degenerate 13-mer motif and those that do not overlap any, and (**c**) those exclusive to testis sample, shared between testis sample and 41 ENCODE cell lines and found in ENCODE cell lines but not in testis. The 95% confidence interval around the mean estimates of recombination rates are shown in shaded colours.

We found male and female recombination rate to be strongly associated with density of H3K4me3 marks (Fig. 6a, Supplementary Fig. 17), and that H3K4me3 marks containing at least one degenerate PRDM9 motif are more recombinogenic in both sexes than those lacking a motif (Fig. 6b). H3K4me3 marks in testis are not all interchangeable to the marks associated with transcription in other tissue. The H3K4me3 marks specific to testis show increased rate in both sexes compared to marks seen in other ENCODE tissues alone (Fig. 6c), while H3K4me3 marks found in other ENCODE cell lines but not in testis show no effect on the recombination rate at all. As such, we predict at least some overlap in H3K4me3 marks between spermatocytes and oocytes during the initialization of recombination, likely due to PRDM9 action.

## Discussion

Recent years have seen great strides in our understanding of the molecular basis of recombination. This has been notably enabled by the availability of fine-scale and genome-wide assays that allow measurement of recombination rates and related factors in humans, mice and other species^1^,^15^,^21^,^22^,^23^,^24^,^25^. However, less progress has been made in understanding the underpinnings of sexual dimorphism in recombination. On one hand, this is because fine scale estimates of recombination are most easily obtained from patterns of LD, which are intrinsically sex averaged. On the other hand, experimental approaches for characterizing meiotic recombination rates tend to be biased towards males, owing to the ability to characterize recombination events or DSBs in sperm. Although small-scale studies have already been conducted^25^,^26^, the difficulty of obtaining sufficient oocytes to characterize large numbers of completed crossover events in females means that such approaches are unlikely to be applied at scale, at least in human, for the foreseeable future.

The huge quantity of human genotyping data in pedigrees now provides an alternative means to study sex-specific patterns of recombination. In this study, we have exploited the large quantity of available data to construct sex-specific genetic maps that begin to approach the resolution of LD-based sex-averaged maps. Our fine-scale map of recombination highlights that a substantial fraction of the human genome recombines at different rates in females and males, and these differences are largely attributable to the fine scale. At this scale, our sex-specific maps reveal two consistent dimorphic patterns, first that THE1B elements are more recombinogenic in males and second, that female recombination drives an elevation of recombination in the promoter region of genes. As our maps are mainly representative of Europeans, it remains to be determined if these dimorphic patterns are found in other populations. Given the fundamental role of recombination in evolution, the observed dimorphism may have implications for understanding the role of recombination in shaping patterns of human genetic diversity. As more data is collected, it will become possible to not only build maps partitioned by sex, but also in terms of other factors known to influence recombination, including both genetic and environmental factors (such as parental age).

## Methods

### Collection of recombination events

We assembled a collection of recombination events inferred from genome-wide SNPs data in human pedigrees comprising a total of 104,246 informative meioses (57,919 female and 46,327 male meioses). This collection is derived from six sets of recombination data, including one that we have previously analysed^2^, as well as five publicly available data sets from previous studies^6^,^7^,^11^,^12^,^13^ (Supplementary Methods).

### Building the recombination maps

The 22 autosomes and X chromosome were split into 833,754 and 18,039 SNP intervals respectively, as defined by the boundaries of all recombination events found in the combined data set. We estimated female and male recombination rates within each of these SNPs intervals. First, we build female and male unrefined maps based on the maximum likelihood estimates of rates (Supplementary Methods). Second, we implemented a MCMC procedure to build the refined maps. We assumed that recombination rate in each interval was independent from every other SNP interval. We modelled the underlying recombination rate within a SNP interval as a random variable drawn from a gamma distribution, with the prior parameters selected via inspection of the unrefined map. We used a Gibbs sampler to iteratively sample the SNP interval location of each recombination event, and then resampled recombination rates consistent with the event assignment. For each SNP interval, we obtained a mean posterior estimate of the recombination rate and the associated CI by running 1.3 million iterations, and discarding the first 300,000 as burn-in. We sampled from the chain every 100 iterations to obtain 10,000 samples. We applied this method separately to female and male recombination events, obtaining separate female and male refined maps. Our method to refine the estimates of recombination rates is available here: github.com/auton1/rMCMC. The recombination maps generated can be downloaded here: https://github.com/cbherer/Bherer_etal_SexualDimorphismRecombination..

### Identifying dimorphic regions of recombination

To identify regions of the genome with significant rate difference between the sexes, we used the CI of posterior recombination rates obtained with our mapping procedure. At the 99% level, we found 6,936 intervals with significantly higher rate in females than males and conversely, 1,427 intervals with significantly higher rates in males than females. To retain only well-localized dimorphic intervals, those smaller than 100 bp (*n*=250) and larger than 10 kb (*n*=475) were excluded. We defined dimorphic regions as 10 kb windows centred around this set of high-confidence and well-localized dimorphic SNPs intervals. Adjacent dimorphic intervals were merged and considered as one larger dimorphic interval. We verified that female recombinogenic regions contained only dimorphic SNP interval with higher rate in females, and conversely for males recombinogenic regions. We excluded 10 kb regions with recombination rate difference between the sexes below 2 cM per Mb. We also excluded regions overlapping gaps, as defined in the UCSC table. In the cases of overlap between regions, we kept the region with highest difference in rate between the sexes.

### Wavelet analysis

We used the estimation of female and male recombination rates from the refined map and interpolated the map in non-overlapping bins of 1 kb across each of the 22 autosomes. Bins were set to `̀NA'' if they overlapped a centromere, gaps in the assembly, or were located in low-resolution regions of our map (where inter-SNPs intervals size>50 kb). For each bin, we also computed a number of genomic and epigenomic annotations, as listed in Supplementary Table 6. GC content and CpG content were computed using the hg19 build of human reference genome. The percentage of bins in CpG islands was computed using the CpG island track from the UCSC Genome Browser. SNP density in Europeans was computed using the 1000 Genomes Phase 3 variants list^16^. We computed the exon percentage as the proportion of each 1 kb bin overlapping transcripts listed in the Gencode gene list (v19). Per cent overlap with a given repeat family or repeat elements were computed using the UCSC repeat masker table. We obtained DNA motif locations using our program (available here: https://github.com/auton1/motiflocation) applied to the reference genome. Per cent overlap with histone peaks was computed using the ENCODE data, as well as H3k4me3 peaks from testis sample kindly provided K. Brick and published in ref. 22.

For the wavelet analysis, each signal was padded with NAs to obtain a number of bins equal to a power of two. As such, any wavelet coefficient influenced by edge effects would also be NA, and hence excluded from analysis. Using each annotation as the signal, we applied a DWT, as implemented in the WMTSA R package, and using the Haar wavelet function to yield detail and smoothing coefficients for scales from 2 to 3,278 kb^27^.

### Data availability

The recombination maps generated in this study are available for download at: https://github.com/cbherer/Bherer_etal_SexualDimorphismRecombination. The refined genetic maps include: the refined maps, the refined European maps, and the refined Icelandic map. For each of them, we share the female, male and sex-averaged estimates of recombination rates.

## Additional information

**How to cite this article:** Bhérer, C. *et al*. Refined genetic maps reveal sexual dimorphism in human meiotic recombination at multiple scales. *Nat. Commun.* **8,** 14994 doi: 10.1038/ncomms14994 (2017).

**Publisher's note**: Springer Nature remains neutral with regard to jurisdictional claims in published maps and institutional affiliations.

## Supplementary Material

Supplementary Information Supplementary Figures, Supplementary Tables, Supplementary Methods, and Supplementary References

## Figures and Tables

**Table 1:** Summary of the recombination data from recent genomic studies of human families.

	**Nb. of meioses**				
**Data set**	**Females**	**Males**	**Total**	**Predominant ancestry**	
Bleazard *et al*.^11^	481	481	962	Asian	
Campbell *et al*.^2^	9,785	9,784	19,591	European	
Fledel-Alon *et al*.^6^	3,787	3,768	7,555	European	
Hinch *et al*.^12^	524	526	1,050	African American	
Kong *et al*.^7^	41,745	30,184	71,929	Icelandic	
Martin *et al*.^13^	1,597	1,584	3,181	European	
**Total**	57,919	46,327	104,246		

## References

[b1] Broman K. W., Murray J. C., Sheffield V. C., White R. L. & Weber J. L. Comprehensive human genetic maps: individual and sex-specific variation in recombination. Am. J. Hum. Genet. 63, 861–869 (1998). 9718341 10.1086/302011 PMC1377399

[b2] Campbell C. L., Furlotte N. A., Eriksson N., Hinds D. & Auton A. Escape from crossover interference increases with maternal age. Nat. Commun. 6, 6260 (2015). 25695863 10.1038/ncomms7260 PMC4335350

[b3] Coop G., Wen X., Ober C., Pritchard J. K. & Przeworski M. High-resolution mapping of crossovers reveals extensive variation in fine-scale recombination patterns among humans. Science 319, 1395–1398 (2008). 18239090 10.1126/science.1151851

[b4] Kong A. . Fine-scale recombination rate differences between sexes, populations and individuals. Nature 467, 1099–1103 (2010). 20981099 10.1038/nature09525

[b5] Kong A. . A high-resolution recombination map of the human genome. Nat. Genet. 31, 241–247 (2002). 12053178 10.1038/ng917

[b6] Fledel-Alon A. . Variation in human recombination rates and its genetic determinants. PLoS ONE 6, e20321 (2011). 21698098 10.1371/journal.pone.0020321 PMC3117798

[b7] Kong A. . Common and low-frequency variants associated with genome-wide recombination rate. Nat. Genet. 46, 11–16 (2014). 24270358 10.1038/ng.2833

[b8] Baudat F. . PRDM9 is a major determinant of meiotic recombination hotspots in humans and mice. Science 327, 836–840 (2010). 20044539 10.1126/science.1183439 PMC4295902

[b9] Stefansson H. . A common inversion under selection in Europeans. Nat. Genet. 37, 129–137 (2005). 15654335 10.1038/ng1508

[b10] Berg I. L. . Variants of the protein PRDM9 differentially regulate a set of human meiotic recombination hotspots highly active in African populations. Proc. Natl Acad. Sci. USA 108, 12378–12383 (2011). 21750151 10.1073/pnas.1109531108 PMC3145720

[b11] Bleazard T., Ju Y. S., Sung J. & Seo J. S. Fine-scale mapping of meiotic recombination in Asians. BMC Genet. 14, 19 (2013). 23510153 10.1186/1471-2156-14-19 PMC3599818

[b12] Hinch A. G. . The landscape of recombination in African Americans. Nature 476, 170–175 (2011). 21775986 10.1038/nature10336 PMC3154982

[b13] Martin H. C. . Multicohort analysis of the maternal age effect on recombination. Nat. Commun. 6, 7846 (2015). 26242864 10.1038/ncomms8846 PMC4580993

[b14] Hussin J., Roy-Gagnon M. H., Gendron R., Andelfinger G. & Awadalla P. Age-dependent recombination rates in human pedigrees. PLoS Genet. 7, e1002251 (2011). 21912527 10.1371/journal.pgen.1002251 PMC3164683

[b15] The International HapMap Consortium. A second generation human haplotype map of over 3.1 million SNPs. Nature 449, 851–861 (2007). 17943122 10.1038/nature06258 PMC2689609

[b16] The 1000 Genomes Project. A global reference for human genetic variation. Nature 526, 68–74 (2015). 26432245 10.1038/nature15393 PMC4750478

[b17] Myers S., Bottolo L., Freeman C., McVean G. & Donnelly P. A fine-scale map of recombination rates and hotspots across the human genome. Science 310, 321–324 (2005). 16224025 10.1126/science.1117196

[b18] Myers S. . Drive against hotspot motifs in primates implicates the *PRDM9* gene in meiotic recombination. Science 327, 876–879 (2010). 20044541 10.1126/science.1182363 PMC3828505

[b19] Spencer C. C. . The influence of recombination on human genetic diversity. PLoS Genet. 2, e148 (2006). 17044736 10.1371/journal.pgen.0020148 PMC1575889

[b20] Myers S., Freeman C., Auton A., Donnelly P. & McVean G. A common sequence motif associated with recombination hot spots and genome instability in humans. Nat. Genet. 40, 1124–1129 (2008). 19165926 10.1038/ng.213

[b21] McVean G. A. . The fine-scale structure of recombination rate variation in the human genome. Science 304, 581–584 (2004). 15105499 10.1126/science.1092500

[b22] Pratto F. . DNA recombination. Recombination initiation maps of individual human genomes. Science 346, 1256442 (2014). 25395542 10.1126/science.1256442 PMC5588152

[b23] Wang J., Fan H. C., Behr B. & Quake S. R. Genome-wide single-cell analysis of recombination activity and *de novo* mutation rates in human sperm. Cell 150, 402–412 (2012). 22817899 10.1016/j.cell.2012.06.030 PMC3525523

[b24] Jeffreys A. J. & May C. A. Intense and highly localized gene conversion activity in human meiotic crossover hot spots. Nat. Genet. 36, 151–156 (2004). 14704667 10.1038/ng1287

[b25] Hou Y. . Genome analyses of single human oocytes. Cell 155, 1492–1506 (2013). 24360273 10.1016/j.cell.2013.11.040

[b26] Ottolini C. S. . Genome-wide maps of recombination and chromosome segregation in human oocytes and embryos show selection for maternal recombination rates. Nat. Genet. 47, 727–735 (2015). 25985139 10.1038/ng.3306 PMC4770575

[b27] Percival D. B. & Walden A. T. Wavelet Methods for Time Series Analysis Cambridge University Press (2000).

