# Supplementary material for: Refined genetic maps reveal sexual dimorphism in human meiotic recombination at multiple scales
Source: Nat Commun. 2017 Apr 25;8:14994. doi: 10.1038/ncomms14994 (PMC5414043; doi:10.1038/ncomms14994)
Supplement: Supplementary Information — Supplementary Figures, Supplementary Tables, Supplementary Methods, and Supplementary References [file ncomms14994-s1.pdf]

Supplementary Methods

1. Data

We used recombination data from six recent studies of human pedigrees, including one that we previously analyzed[1] and five datasets released publicly. Table 1 presents these datasets with their associated number of female and male meioses.

1.1. Campbell 2015 dataset

We used a large-scale dataset of recombination events derived from collaboration with the direct-to-consumer genetic testing company, 23andMe Inc. (Mountain View, CA). Our dataset consists of 4,470 pedigrees contributing a total of 19,569 informative meioses, of which 9,785 are from females and 9,784 are from males, of which we had previously analyzed 4,209 pedigrees[1]. Recombination events were inferred within nuclear families using the Lander-Green algorithm implemented in Merlin (version 1.1.2)[2]. After applying filtering steps described in Campbell *et al*., 2015, the filtered dataset consists of 685,907 recombination events (Supplementary Table 2).

1.2. Publicly available datasets

We collected publicly available datasets of recombination events from five sources. The first source is Bleazard *et al*. 2011[3], who published an Asian map based on 962 meioses in Mongolians and Koreans nuclear families with equal number of female and male transmissions. Our second source is Hinch *et al*. 2011[4], who provided us filtered recombination events from 524 female and 526 male transmissions in African-American pedigrees (Jackson Heart Study and Cleveland Family Study as well as a study at the Children's Hospital of Philadelphia). The third source is Kong *et al*. 2014[5] who released 2.26 million recombination events from 41,745 female and 30,184 male meioses genotyped in the deCODE Genetics database. Importantly, this dataset does not provide information on subtelomeric regions (average length ~5Mb), where the Kong *et al*. method for calling recombination events is less reliable. The fourth set of recombination events was made available by Fledel-Alon *et al*. in 2011[6], and are pertaining to 4,160 meioses (2,083 female and 2,077 male) from the Framingham Heart Cohort Study, 1,881 meioses (947 female and 934 male) from the Autism Genetic Resource Exchange and 1,514 meioses (757 female and 757 male) from the Hutterite population, all deriving from parents of European origins based on PCA analysis. The fifth source is Martin *et al*.[7], who inferred recombination events in eight family cohorts comprising: French Canadian pedigrees[8], the INGI-CARL Cohort from Carlantino, the INGI-FVG Cohort from Friuli Venezia Giulia, the INGI-VAL Cohort from Val Borbera, the Orkney Complex Disease Study, the Uganda General Population Cohort, the Netherlands Twin Registry and the Queensland Twin Registry. This last dataset provided recombination events for 1,597 female and 1,584 male informative meioses.

1.3. Liftover

We used UCSC Genome Browser liftOver tool to convert the recombination events coordinates from earlier genome builds to build hg19. We verified that no events switched to other chromosomes or had flipped start and end positions between builds. We excluded events that could not be mapped unambiguously and those with a size change larger than 1kb. Less than 3% of events could not be mapped unambiguously to build hg19 (Supplementary Table 3).

2. Building the genetic maps

2.1. Unrefined maps

For each interval *i* spanned by two adjacent SNPs, let λ_i denote the probability of a recombination event in a meiosis. If a recombination event could always be assigned precisely to an interval spanned by two adjacent SNPs, the estimate of λ_i would be the number of recombination events observed in that interval divided by total number of meioses studied. However, recombination events are generally assigned to regions spanning more than one inter-SNP interval. Therefore, for a recombination event attributed to a region that spanned *k* equally-spaced intervals, only an expected count of 1/*k* can be assigned to each interval. Tabulated over *m* meiosis, the sum of expected count attributed to interval *i* divided by *m*, i.e. the recombination fraction, is the estimate of λ_i. For SNPs intervals that are unevenly spaced, the expected count is computed as the proportional contribution of each interval to each event, which is the size ratio of the interval over the event.

Using the combined dataset of 104,246 meioses, we constructed unrefined genetic maps for females and males separately. We used the Kosambi function to convert recombination fraction to genetic distances. The number of meioses was specified in each inter-SNPs intervals in order to account for the reduced number of meioses at the chromosome ends arising from the deCODE events having been truncated at chromosome ends[9]. To build the sex-averaged genetic maps, we interpolated and averaged the female and male rates in each interval spanned by the union of SNPs in the sex-specific maps. The resolution of the unrefined maps is limited by the size at which recombination events could be resolved in the original datasets, which ranges from 28kb to 105kb (Table 1). This is greater than the size of a typical hotspot, which is 1-2kb. Importantly, in the unrefined maps, the estimation of recombination rates does not take into account the overlap of events.

2.2. Refined maps

To improve localization of events, we implemented a Bayesian MCMC procedure that takes advantage of the knowledge that rates are shared across individuals of a given sex and that recombination occurs in hotspots. Our approach is similar to a previous method applied to recombination events detected from patterns of admixture[4], although our model is suitable for recombination events called in pedigrees rather than from ancestry changepoints. This allows us to compute an approximate posterior distribution of recombination rates for each interval *i*, for females and males.

In the collection of datasets, we defined a total of 833,754 intervals for the autosomes using the union of SNPs defining the boundaries of female and male recombination events. We modeled the genome as a sequence of SNP intervals with independent recombination probabilities shared by a group of individuals. We assumed the recombination fraction in each interval to be a gamma distributed random variable. To take into account the long-range correlation between recombination rates along the genome, we impose a prior variance of recombination rate that is consistent with empirical estimations from LD-based map (see below). Using the same model, we also computed the X chromosome female genetic map using 18,039 intervals and 85,331 female events.

The MCMC proceeds as follow:

1. Step 0: Starting state of the MCMC

We initialize the Gibbs sampler by sampling recombination fraction lambdas for each interval from a prior Gamma distribution described below.

2. Step 1: Sampling the locations of recombination events

In this step, we sample the SNP interval locations of each recombination event. Specifically, we sample the SNPs interval in which a recombination event occurred conditional on the vector of recombination fractions $\bar{\lambda}$ sampled at the previous state. Let a recombination event x span SNP intervals from j to k. We sample a SNP interval location for this event with probability

$$\begin{cases} \dfrac{\lambda_i}{\sum_{m=j}^{k} \lambda_m} & \text{if } k \geq i \geq j, \\ 0 & \text{otherwise.} \end{cases}$$

We sample the SNPs intervals in which each event is placed for all events, and count the number of events, n_i, placed into each SNPs interval i.

3. Step 2: Sampling the recombination rates in each sequence interval

In this step, we sample the recombination fractions, $\bar{\lambda}$, from an approximate posterior distribution conditional on the events sampled in step 1. In each interval, we model the number of recombination events as following a Poisson distribution with parameter $\lambda_i m$, where m is the number of meioses.

$$n_i \sim Poisson(\lambda_i m)$$

We use an independent Gamma prior on recombination fractions with shape parameter α_i and inverse scale parameter β_i. If the mean of the gamma distribution is μ_i and the variance v_i, then, the parameters of the distribution are estimated to be $\alpha_i = {\mu_i}^2/v_i$ and $\beta_i = \mu_i/v_i$. To set the parameters of the prior distribution for each SNPs interval, we set the mean recombination rate equal to the chromosome mean estimate from the unrefined map. We choose an uninformative prior variance, and for each SNP interval we set the variance as function of the SNP interval size, increasing proportional to the size of the interval to the power 1.65. As such, we have:

$$\lambda_i \sim Gamma(\alpha_i, \beta_i)$$

Rescaling with the number of meioses, m:

$$m\lambda_i \sim Gamma(\alpha_i, \frac{1}{m}\beta_i)$$

Using this Gamma prior, we obtain a Gamma posterior by the property of conjugacy:

$$\lambda_i \mid n_i \sim Gamma(\alpha_i + n_i,\ \beta_i + m)$$

Having obtained the posterior distribution, we return to step 1.

We ran 1.3 million iterations for each chromosome. We removed the first 300,000 iterations as burn-in. We computed the mean posterior estimate of recombination rate in each interval using the 100th sample. Recombination fractions were converted into recombination rates in cM/Mb using the Kosambi function. We also computed the variance, 95% and 99% credible intervals (CI) of the posterior samples. The latter were used to define the sex-dimorphic regions of recombination in the human genome.

Supplementary Tables

Supplementary Table 1. Description of the parental ancestry of each meiosis within the combined dataset

	Females		Males		Both sexes	
**Ancestry**	**Count**	**%**	**Count**	**%**	**Count**	**%**
European	54,629	94.3	43,094	93.0	97,723	93.7
East Asian	999	1.7	902	1.9	1,901	1.8
African American	856	1.5	828	1.8	1,684	1.6
Latino	816	1.4	761	1.6	1,577	1.5
None/other	301	0.5	392	0.8	693	0.7
South Asian	208	0.4	203	0.4	411	0.4
Middle Eastern	110	0.2	147	0.3	257	0.2
**Total**	**57,919**	**100**	**46,327**	**100**	**104,246**	**100**

Supplementary Table 2. Recombination events in the original datasets

Dataset	Nb. of recombination events				Build	Reso-lution*	Calling method
	Females		Males	Total			
	Autosomes	ChrX					
Bleazard 2013	19,232	855	12,835	32,922	36	105.2	HMM[3]
Campbell 2015	408,171	17,519	260,217	685,907	37	27.8	Merlin[2]
Fledel-Alon 2011	157,838	0	101,974	259,812	36	89.6	Heuristic method[10]
Hinch 2011	19,194	0	12,647	31,841	36	38	HMM[4]
Kong 2014	1,614,595	68,758	580,970	2,264,323	36	38.6	Long-range phasing[11]
Martin 2015	66,707	0	42,305	109,012	36 / 37	38.2	duoHMM[12]
**Total**	**2,285,737**	**87,132**	**1,010,948**	**3,383,817**		37.4	

* Resolution: median size of autosomal events

Supplementary Table 3. Number of recombination events in the combined dataset in build hg19

Dataset	Nb. of recombination events				
	Females			Males	Total
	Autosomes	ChrX	Sub-total		
Bleazard 2013	18,839	813	19,652	12,502	32,154
Campbell 2015	408,171	17,519	425,690	260,217	685,907
Fledel-Alon 2011	154,680	0	154,680	99,517	254,197
Hinch 2011	19,031	0	19,031	12,469	31,500
Kong 2014	1,586,588	66,999	1,653,587	572,529	2,226,116
Martin 2015	65,988	0	65,988	41,773	107,761
**Total**	**2,253,297**	**85,331**	**2,338,628**	**999,007**	**3,337,635**

Supplementary Table 4. Number of sex-specific hotspots inferred in males and females given different criteria for defining a 10kb dimorphic region as being a hotspot in one sex and a coldspot in the other sex. Results reported in the main text are for sex-specific hotspots defined as a 10 kb dimorphic region with mean rate > 10 cM/Mb in one sex and peak rate across all intervals overlapping the region < 1 cM/Mb in the other sex.

Hotspot /coldspot criterion		Count of sex-specific hotspots	
Hot sex	Cold sex	Females	Males
Mean rate > 10 cM/Mb	Peak rate < 1 cM/Mb	304	147
Mean rate > 10 cM/Mb	Peak rate < 3 cM/Mb	601	256
Mean rate > 5 cM/Mb	Peak rate < 1 cM/Mb	922	253
Mean rate > 10 cM/Mb	Mean rate < 1 cM/Mb	682	260
Mean rate > 10 cM/Mb	Mean rate < 3 cM/Mb	1,477	436
Mean rate > 5 cM/Mb	Mean rate < 1 cM/Mb	1,716	425

Supplementary Table 5. Correlation between detail coefficients from Haar discrete wavelet transform of GC-content and Log_{10} recombination rates

	Female		Male		Sex-averaged	
Scale (kb)	R-squared	p-value	R-squared	p-value	R-squared	p-value
2	0.00015	2.8E-43	2.2E-05	1.2E-07	0.00013	9.4E-39
4	0.00086	3.7E-122	0.00013	3.9E-20	0.00074	9.7E-106
8	0.0037	2.7E-256	0.00054	3.6E-39	0.0030	6.3E-214
16	0.0091	7.8E-318	0.0012	1.3E-43	0.0073	4.6E-256
32	0.019	Inf	0.0026	2.1E-47	0.015	2.0E-269
64	0.048	Inf	0.011	4.5E-99	0.043	Inf
128	0.10	Inf	0.033	3.2E-144	0.099	Inf
256	0.18	Inf	0.064	2.2E-136	0.18	Inf
512	0.23	7.1E-245	0.11	2.7E-108	0.23	3.9E-249
1024	0.20	3.2E-94	0.07	3.3E-32	0.19	2.7E-89
2048	0.15	1.7E-29	0.02	1.8E-05	0.13	1.4E-25
4096	0.21	1.4E-14	0.04	0.0017	0.20	3.2E-14
8192	0.18	1.2E-03	0.22	0.00025	0.30	9.4E-06
16384	0.59	3.1E-02	0.47	0.060	0.69	0.016

Supplementary Table 6. Annotations used in the wavelet analysis

Annotation	Source (table or program)	Computation for wavelet analysis	Source
exons	wgEncodeGencodeBasicV19.txt.gz	Percentage of 1kb bins overlapping any coding transcript	http://hgdownload.soe.ucsc.edu/goldenPath/hg19/database/
GC-content	Human reference genome hg 19	Percent GC in 1kb bins	ftp://hgdownload.cse.ucsc.edu/goldenPath/hg19/bigZips/
CpG-content	Human reference genome hg19	Percent CpG in 1 kb bins	ftp://hgdownload.cse.ucsc.edu/goldenPath/hg19/bigZips/
CpG islands	cpgIslandExt.txt.gz	Percentage of 1kb bins overlapping CpG islands	ftp://hgdownload.cse.ucsc.edu/goldenPath/hg19/database/cpgIslandExt.txt.gz
**DNA motif locations**	Human reference genome hg19 and the following motif:	Percentage of 1kb bins overlapping predicted DNA motif locations	ftp://hgdownload.cse.ucsc.edu/goldenPath/hg19/bigZips/
Degenerate 13-mer	CCNCCNTNNCCNC		
Extended 13-mer	CCTCCCTNNCCAC		
Core 7-mer	CCTCCCT		
SNP density in the map		Number of SNPs in the recombination data per 1kb bins	
SNP density 1000 Genome Phase 3	ALL.wgs.phase3_shapeit2_mvncall_integrated_v5a.20130502.sites.vcf.gz	In each 1kb, number of segregating sites in Europeans from the 1000 Genomes Phase 3 release	ftp://ftp.1000genomes.ebi.ac.uk/vol1/ftp/release/20130502/ALL.wgs.phase3_shapeit2_mvncall_integrated_v5a.20130502.sites.vcf.g
Repeat elements	rmsk.txt.gz	Percent overlap btw 1 kb bins and given repeat element	ftp://hgdownload.cse.ucsc.edu/goldenPath/hg19/database/rmsk.txt.gz
**Histone Marks**			
H3k4me3 testis	AB1_H3K4me3_peaks.ENCODE_ol1Kb.bed	Percent overlap btw 1kb and histone peaks	Peaks table provided by Brick. Ref: dePratto et al., 2014
H3K4me3 ENCODE	Selected tables	Percent overlap btw 1kb and histone peaks	http://ftp.ebi.ac.uk/pub/databases/ensembl/encode/integration_data_jan2011/byDataType/peaks/jan2011/histone_macs/optimal/
**DNA methylation**			Guo et al., 2015
CpG methylation - female 17 weeks	GSE63818_PGC_17W_embryo1_F_methylation_calling.bed.gz	Average CpG methylation levels in 1kb bins	
CpG methylation - male 19 weeks - embryo 1	GSE63818_PGC_19W_embryo1_M_methylation_calling.bed.gz	Average CpG methylation levels in 1kb bins	
CpG methylation - male 19 weeks - embryo 2	GSE63818_PGC_19W_embryo2_M_methylation_calling.bed.gz	Average CpG methylation levels in 1kb bins	

Supplementary Figures

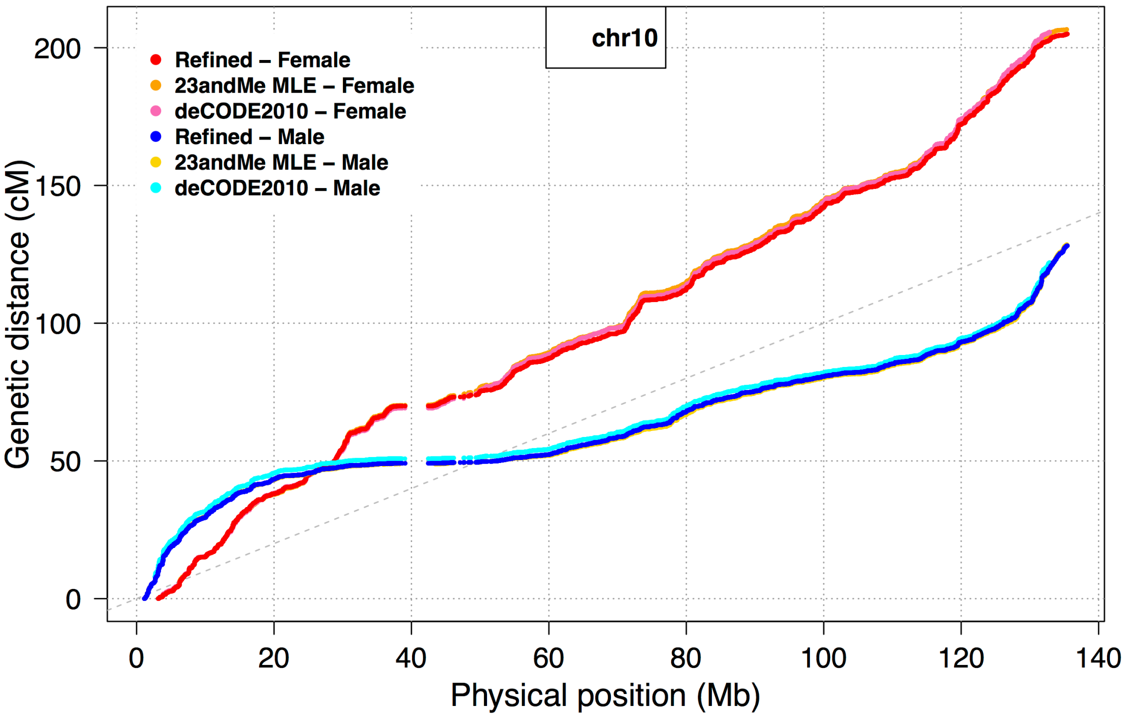

Supplementary Figure 1. Agreement between sex-specific genetic maps. Chromosome 10 is shown as an example. Our refined maps are shown in red and blue for female and male respectively.

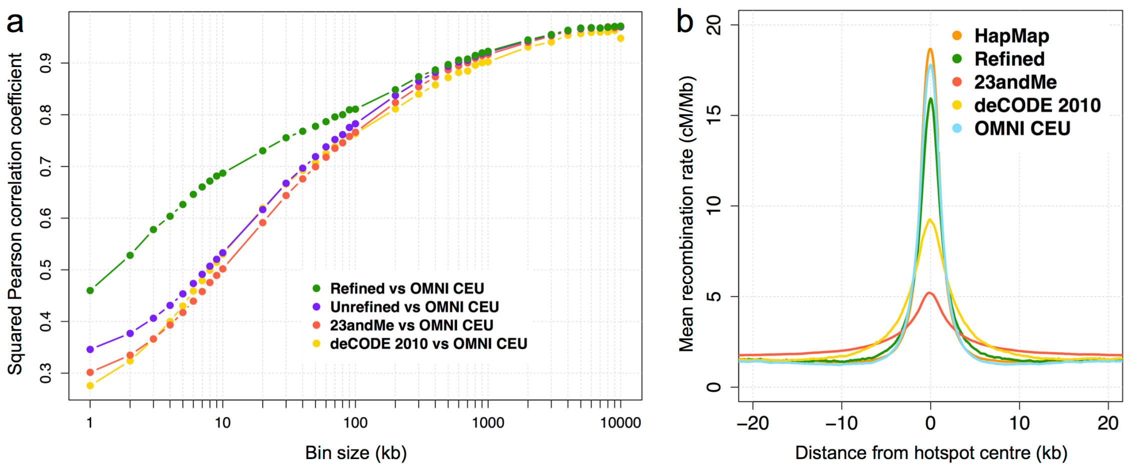

Supplementary Figure 2. Comparison between the refined map and previously generated maps. (a) Squared Pearson correlation as a function of scale between sex-averaged maps and the 1000 Genomes OMNI map based on patterns of linkage disequilibrium in CEU sample. **(b)** Mean recombination rate around hotspots defined in the LD-based HapMap map for a number of sex-averaged maps.

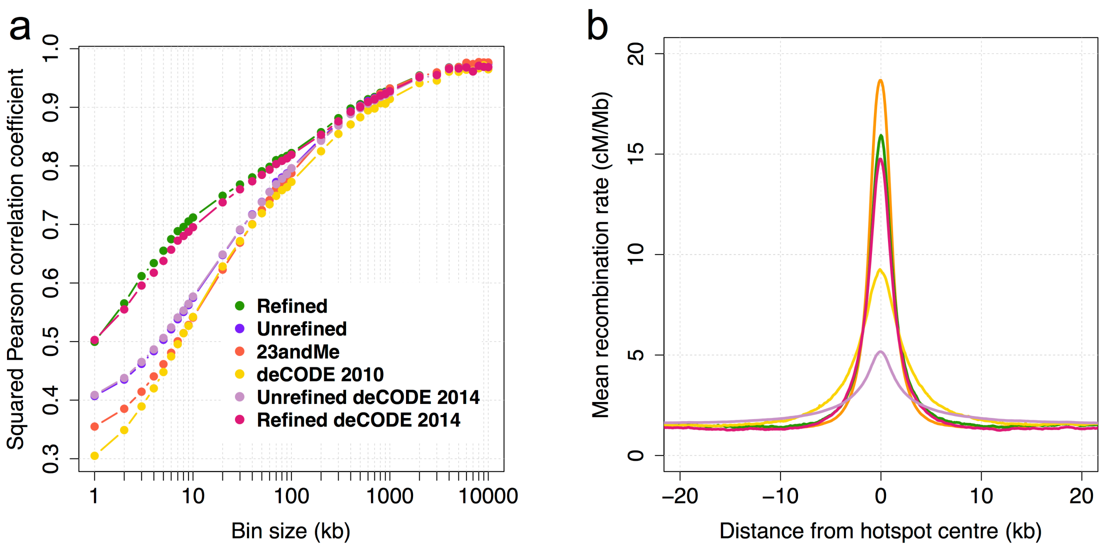

Supplementary Figure 3. Comparison between the deCODE 2014 unrefined and refined and other maps. Refined and unrefined deCODE 2014 maps were constructed using data from Kong et al., 2014 exclusively. In this dataset, recombination data is lacking 5 Mb from each chromosome ends. The correlation shown here excludes these regions for all maps. **(a)** Squared Pearson correlation as a function of scale between sex-averaged maps and the HapMap map. **(b)** Mean recombination rate around hotspots defined in the LD-based HapMap map for a number of sex-averaged maps.

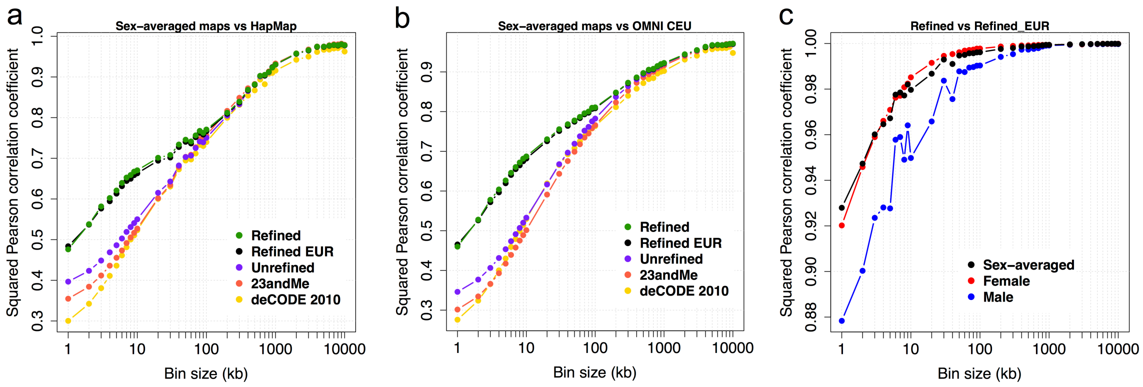

Supplementary Figure 4. Correlation between the refined maps, the European maps, and previously generated maps. The squared Pearson correlation is shown as a function of scale between **(a)** sex-averaged maps and the HapMap map, **(c)** sex-averaged maps and the 1000 Genomes OMNI map based on patterns of linkage disequilibrium in CEU sample, and **(c)** the refined and European sex-specific maps.

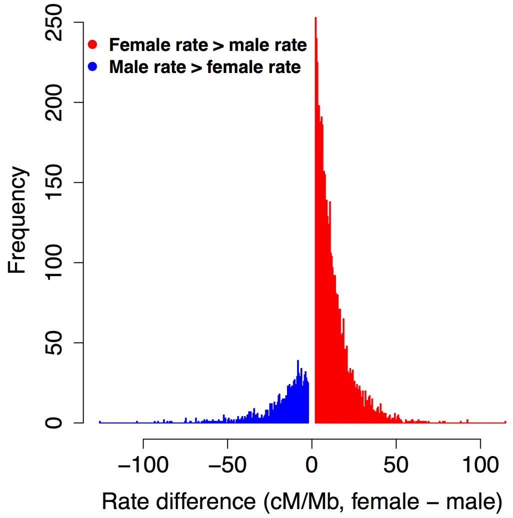

Supplementary Figure 5. Recombination rate difference (female – male) in dimorphic regions. Histograms of the rate differences for female recombinogenic regions (red) and male recombinogenic regions (blue).

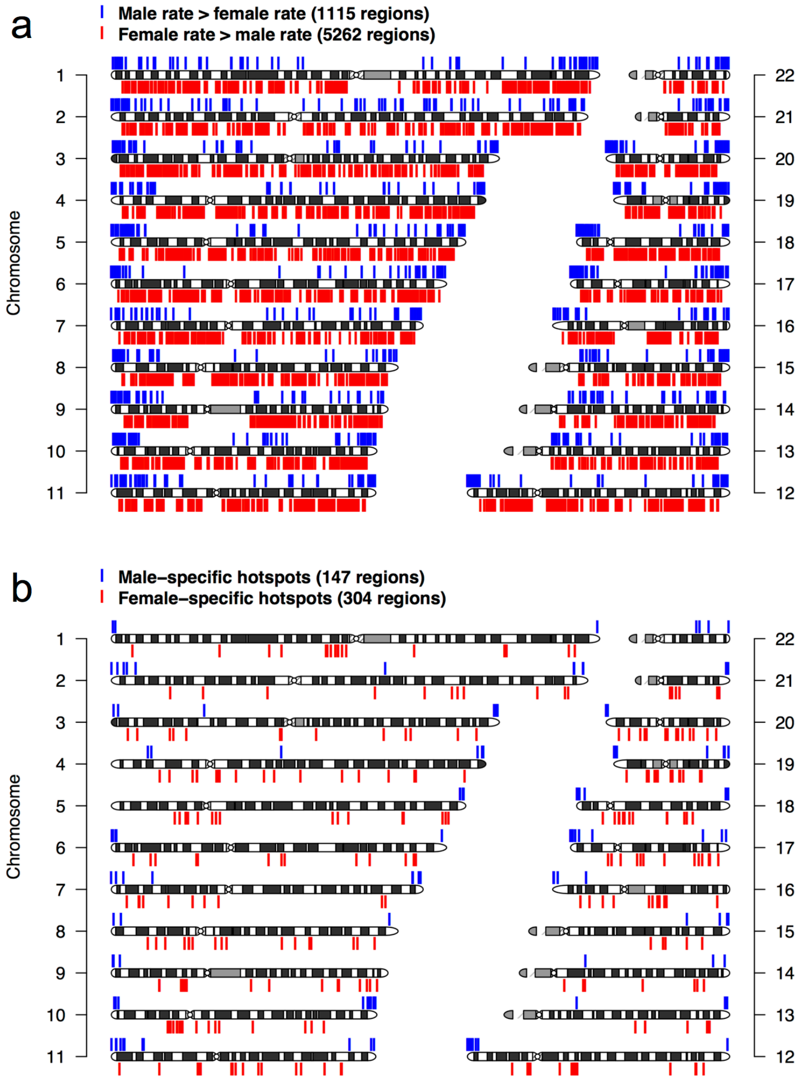

Supplementary Figure 6. Chromosome ideograms representing locations of (a) regions of dimorphic recombination and (b) sex-specific hotspots.

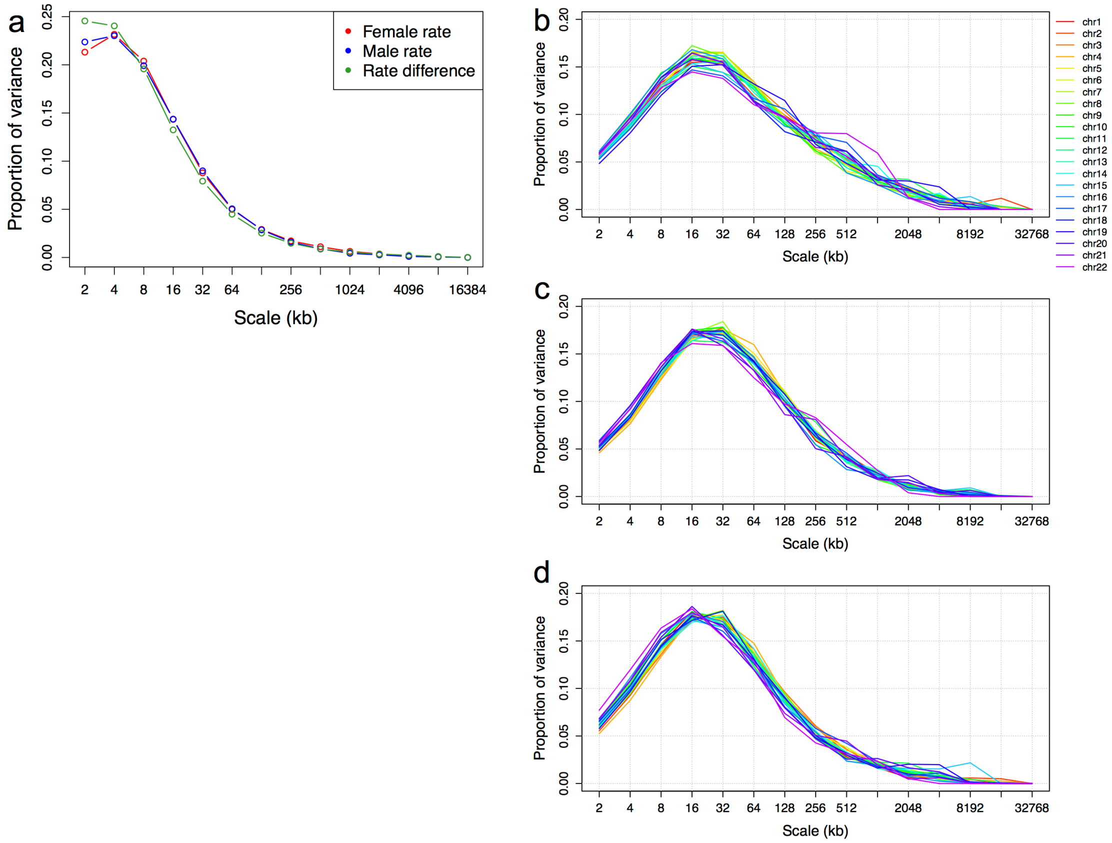

Supplementary Figure 7. Power spectra of the DWT applied to the recombination rates. (a) Genome-wide power spectrum of the raw (not log-transformed) recombination rates. **(b)** Per chromosome power spectrum of the log-transformed female recombination rates, **(c)** male recombination rate and **(d)** recombination rate difference between females and males.

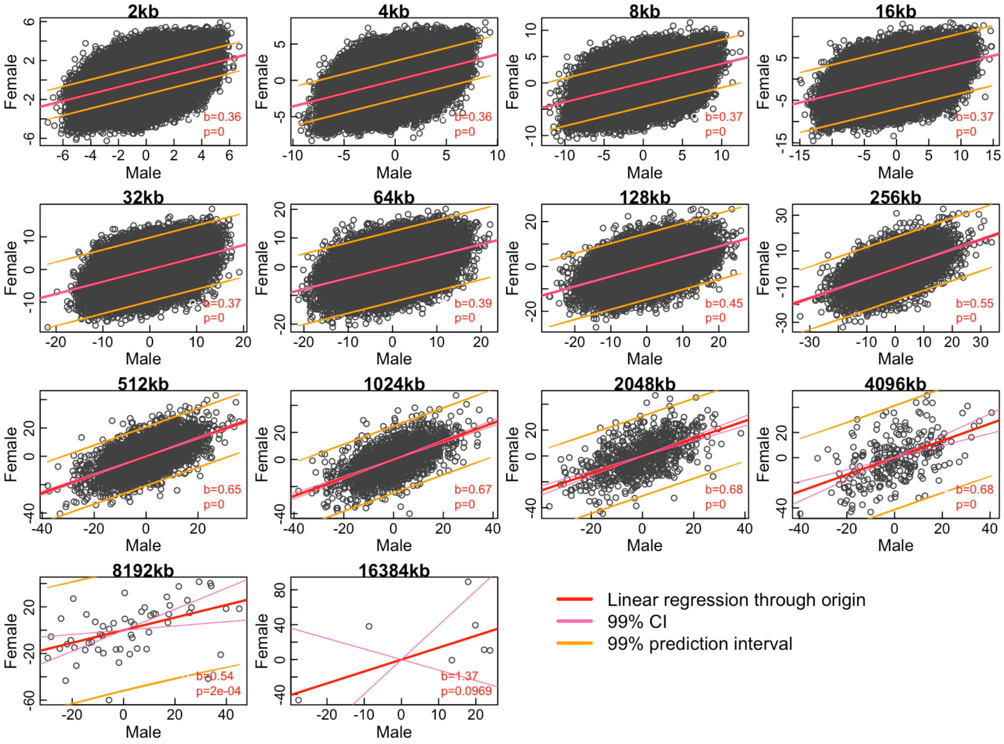

Supplementary Figure 8. Relationship between female and male wavelet transformations. The genome-wide detail coefficients of the discrete wavelet transformation of female and male recombination rates are shown at each scale.

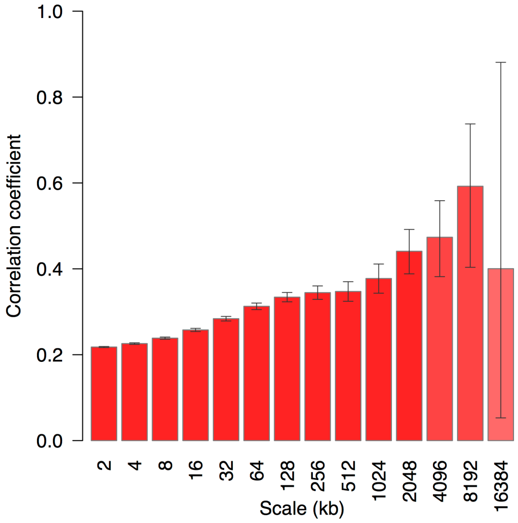

Supplementary Figure 9. Genome-wide correlation between the female and male smooth coefficients as a function of scale. Shown are the squared Pearson correlation coefficients between the smooth coefficients of the discrete wavelet transformation of the female and male log-transformed recombination rates computed at each scale over the 22 autosomes. The color of each bar indicates the p-value of the correlation, with larger values shown in red shades. Error bars represent the 95% confidence interval.

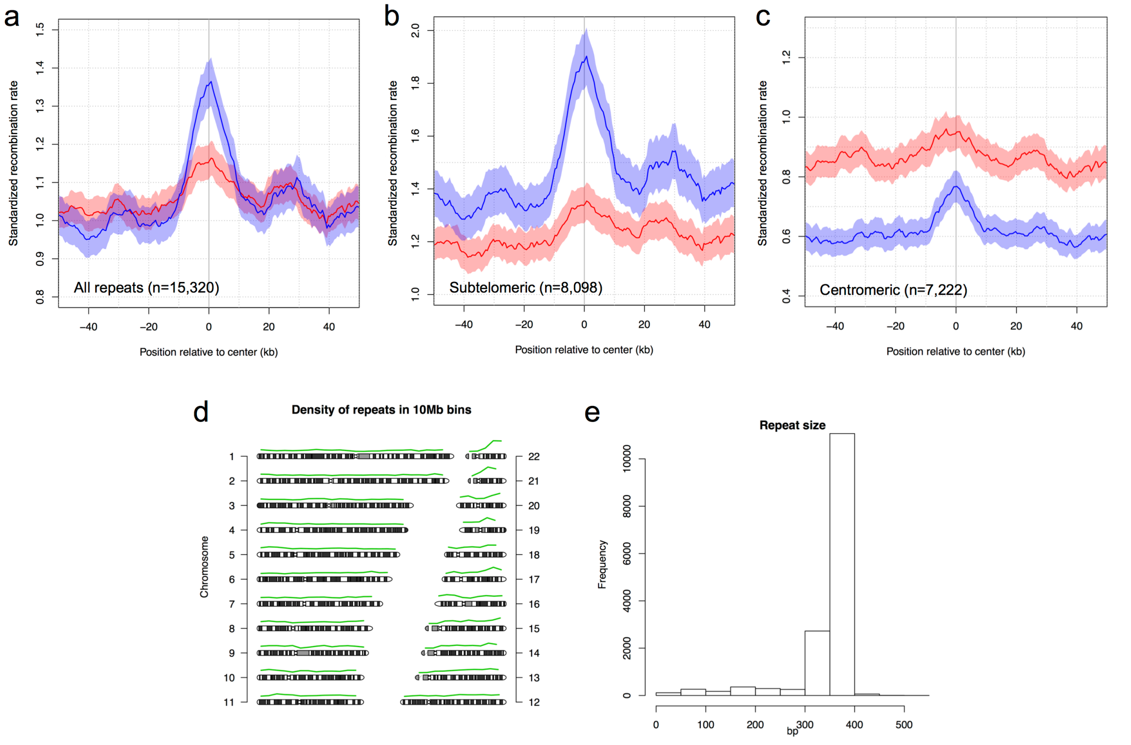

Supplementary Figure 10. Recombination rates around THE1B repeat elements. (a) Average recombination rates around the middle position of a subset of THE1B elements, randomly selected to be spaced by more than 10kb (n=15,320 elements), and **(b)** around selected THE1B elements located in subtelomeric regions (defined as the 1st and last fourth of autosomes) and **(c)** centromeric regions (defined as the 2nd and 3rd fourth of autosomes). **(d)** Density of THE1B elements in 10Mb bins for 22 autosomes. **(e)** Histogram of size of the selected repeats. The 95% confidence interval around the mean estimates of recombination rates are shown in shaded colors.

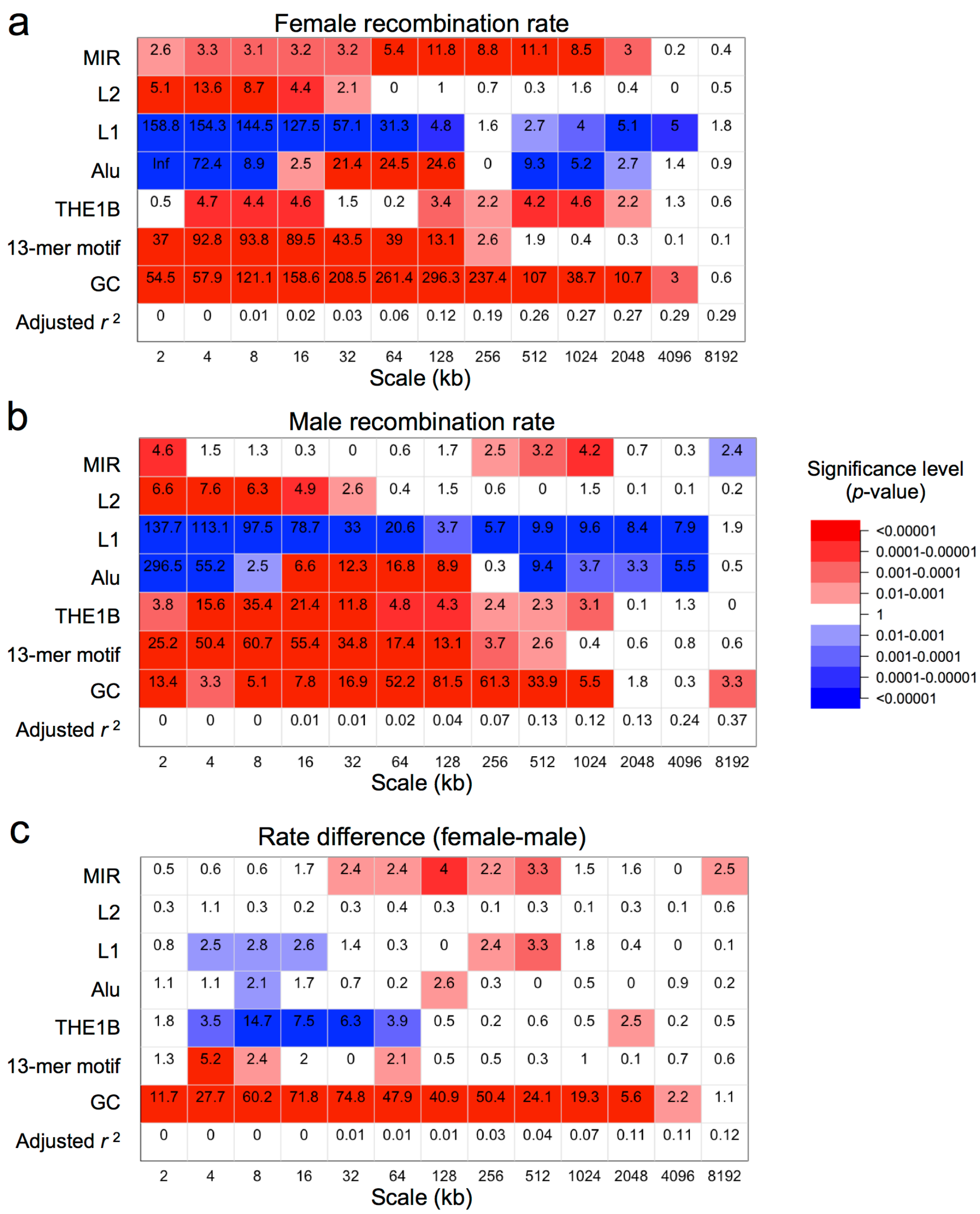

a Female recombination rate

	2	4	8	16	32	64	128	256	512	1024	2048	4096	8192
MIR	2.6	3.3	3.1	3.2	3.2	5.4	11.8	8.8	11.1	8.5	3	0.2	0.4
L2	5.1	13.6	8.7	4.4	2.1	0	1	0.7	0.3	1.6	0.4	0	0.5
L1	158.8	154.3	144.5	127.5	57.1	31.3	4.8	1.6	2.7	4	5.1	5	1.8
Alu	Inf	72.4	8.9	2.5	21.4	24.5	24.6	0	9.3	5.2	2.7	1.4	0.9
THE1B	0.5	4.7	4.4	4.6	1.5	0.2	3.4	2.2	4.2	4.6	2.2	1.3	0.6
13-mer motif	37	92.8	93.8	89.5	43.5	39	13.1	2.6	1.9	0.4	0.3	0.1	0.1
GC	54.5	57.9	121.1	158.6	208.5	261.4	296.3	237.4	107	38.7	10.7	3	0.6
Adjusted r^2	0	0	0.01	0.02	0.03	0.06	0.12	0.19	0.26	0.27	0.27	0.29	0.29

Scale (kb)

b Male recombination rate

	2	4	8	16	32	64	128	256	512	1024	2048	4096	8192
MIR	4.6	1.5	1.3	0.3	0	0.6	1.7	2.5	3.2	4.2	0.7	0.3	2.4
L2	6.6	7.6	6.3	4.9	2.6	0.4	1.5	0.6	0	1.5	0.1	0.1	0.2
L1	137.7	113.1	97.5	78.7	33	20.6	3.7	5.7	9.9	9.6	8.4	7.9	1.9
Alu	296.5	55.2	2.5	6.6	12.3	16.8	8.9	0.3	9.4	3.7	3.3	5.5	0.5
THE1B	3.8	15.6	35.4	21.4	11.8	4.8	4.3	2.4	2.3	3.1	0.1	1.3	0
13-mer motif	25.2	50.4	60.7	55.4	34.8	17.4	13.1	3.7	2.6	0.4	0.6	0.8	0.6
GC	13.4	3.3	5.1	7.8	16.9	52.2	81.5	61.3	33.9	5.5	1.8	0.3	3.3
Adjusted r^2	0	0	0	0.01	0.01	0.02	0.04	0.07	0.13	0.12	0.13	0.24	0.37

Scale (kb)

c Rate difference (female-male)

	2	4	8	16	32	64	128	256	512	1024	2048	4096	8192
MIR	0.5	0.6	0.6	1.7	2.4	2.4	4	2.2	3.3	1.5	1.6	0	2.5
L2	0.3	1.1	0.3	0.2	0.3	0.4	0.3	0.1	0.3	0.1	0.3	0.1	0.6
L1	0.8	2.5	2.8	2.6	1.4	0.3	0	2.4	3.3	1.8	0.4	0	0.1
Alu	1.1	1.1	2.1	1.7	0.7	0.2	2.6	0.3	0	0.5	0	0.9	0.2
THE1B	1.8	3.5	14.7	7.5	6.3	3.9	0.5	0.2	0.6	0.5	2.5	0.2	0.5
13-mer motif	1.3	5.2	2.4	2	0	2.1	0.5	0.5	0.3	1	0.1	0.7	0.6
GC	11.7	27.7	60.2	71.8	74.8	47.9	40.9	50.4	24.1	19.3	5.6	2.2	1.1
Adjusted r^2	0	0	0	0	0.01	0.01	0.01	0.03	0.04	0.07	0.11	0.11	0.12

Scale (kb)

Supplementary Figure 11. Repeat elements associated with female and male recombination rates. Wavelet-based linear model of scale-specific correlates to the **(a)** female recombination rate, **(b)** male recombination rate and **(c)** recombination rate difference between female and male. Tables show the marginal significance (-$\log_{10}$ p-value two-sided t-test) for the linear model analyses of wavelet detail coefficients. Color indicates the direction of the relationship (red=positive; blue=negative) with intensity proportional to significance.

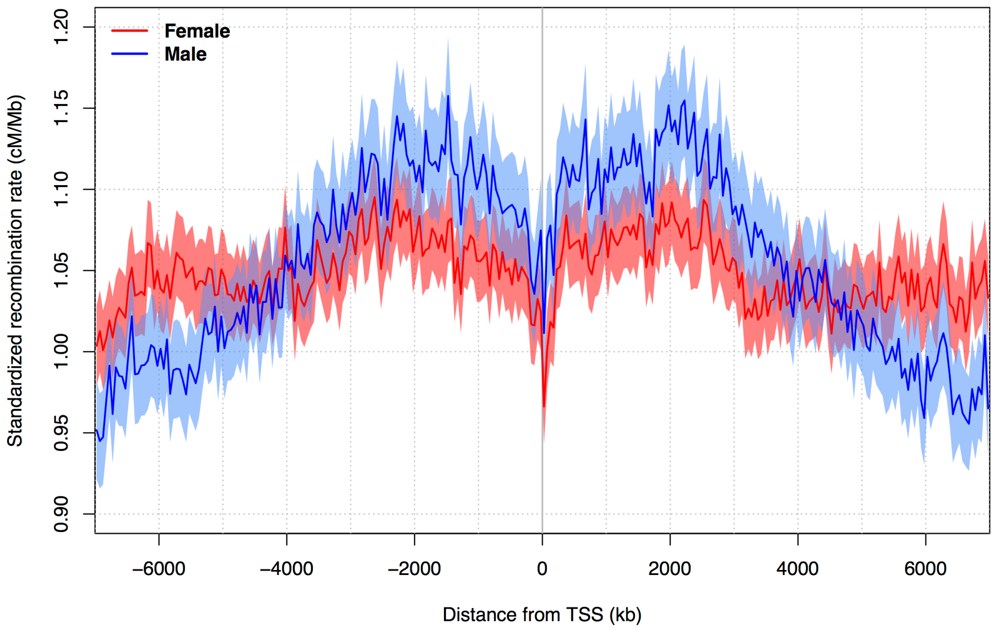

Supplementary Figure 12. Recombination rates megabases away from TSS. Average recombination rate around the transcription start site (TSS) of a subset of 15,239 Gencode genes selected to be spaced to each other by 5kb or more. Rates were interpolated in 50kb bins for 6Mb upstream and downstream of TSS. For each sex, standardized recombination rates were computed in bins as the genetic distance divided by the total genetic distance. The 95% confidence interval around the mean estimates of recombination rates are shown in shaded colors.

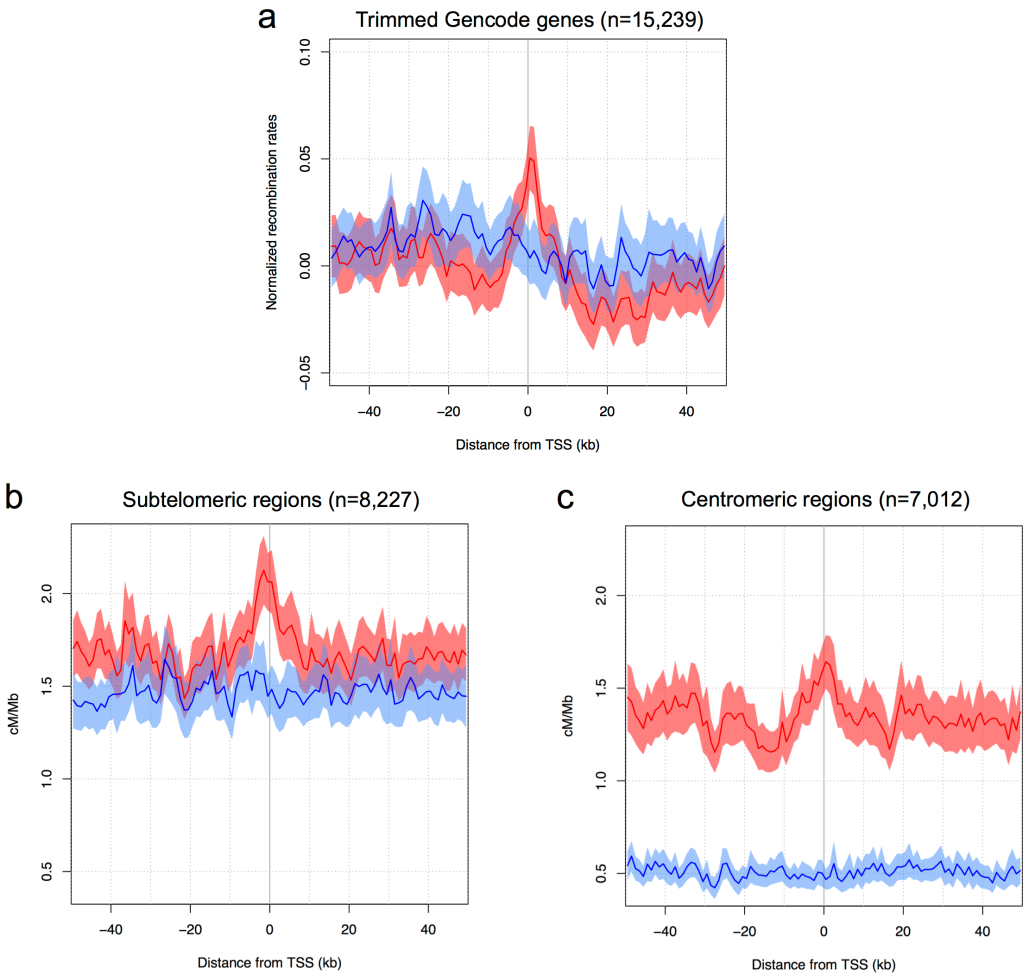

Supplementary Figure 13. Recombination rates across TSS of Gencode genes. Shown are average **(a)** normalized recombination rates and **(b-c)** recombination rates for female (red) and male (blue) across the transcription start site (TSS) of a subset of 15,239 Gencode genes randomly selected to be spaced to each other by 5kb or more. We divided each chromosome into four parts to partition genes into (b) subtelomeric and (c) centromeric location. Rates were interpolated in 1kb bins in a region spanning 50kb upstream and downstream of TSS. The 95% confidence interval around the mean estimates of recombination rates are shown in shaded colors.

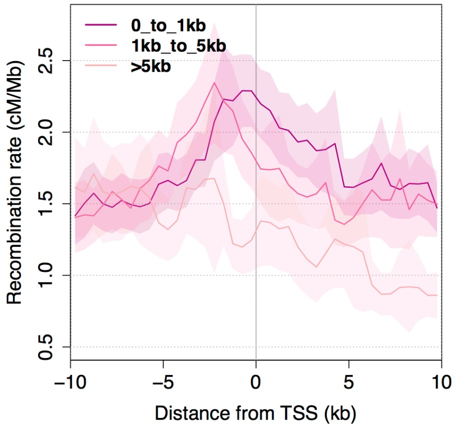

Supplementary Figure 14. Female recombination rates at TSS partitioned by distance to nearest motif. Average female recombination rates were computed in 500bp bins around 7,689 TSS having the nearest motif located upstream. The 95% confidence interval around the mean estimates of recombination rates are shown in tinted colors.

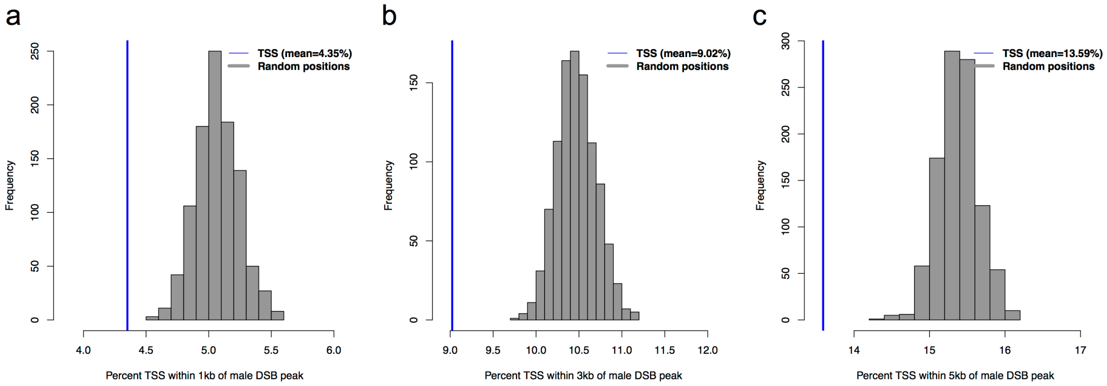

Supplementary Figure 15. Percent overlap of TSS with peaks of DNA double-strand breaks (DSBs) plotted by comparison to distribution of overlap for sets of 1,000 random positions. Shown is percent overlap TSS of a subset of 15,239 Gencode genes trimmed to be spaced to each other by 5kb or more. To draw random positions we 'moved' the TSS positions at a random distance with mean of 50kb, thus conserving the per-chromosome and genomic context distributions. We show the percent overlap within **(a)** 1kb, **(b)** 3kb and **(c)** 5kb.

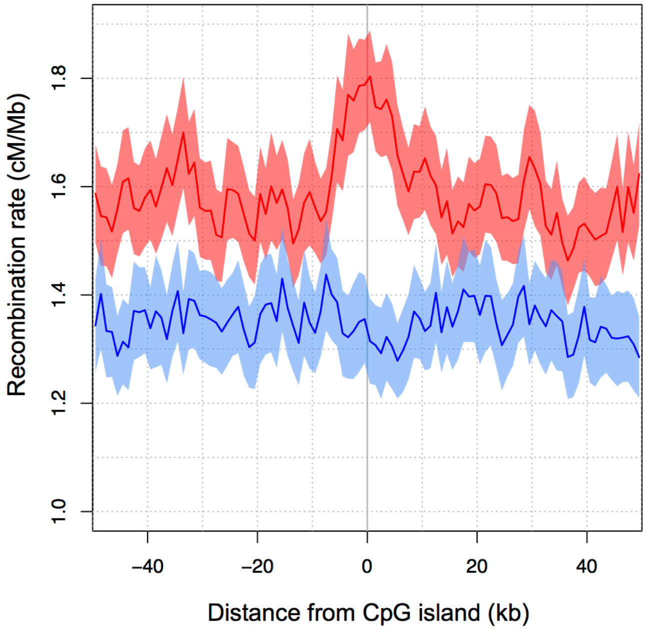

Supplementary Figure 16. Recombination rates around CpG islands. Shown are average female (red) and male (blue) recombination rates across CpG islands. The 95% confidence interval around the mean estimates of recombination rates are shown in shaded colors. Rates were interpolated in 1kb bins in a region spanning 50kb upstream and downstream of middle position of CpG islands.

a Female recombination rate

	2	4	8	16	32	64	128	256	512	1024
Male DNA methylation	11.1	16	12.5	12.7	2.6	1	0.7	0.6	1.2	0.6
Female DNA methylation	30.2	31.3	21	7.5	4.3	1.6	0.3	0.5	1.2	0.3
H3K4me3	Inf	Inf	Inf	219.6	75.3	23.1	6.2	1.3	0.3	0.3
CpG islands	15.1	43.3	64.2	44.3	14.6	13.5	2	1.5	0.1	0.3
CpG-content	8.9	2.6	0.2	0.5	0.5	0.4	0.8	0.9	1.2	0.7
GC-content	1.5	2.3	0.3	4.9	9.8	21.8	30.8	19.9	7.5	1.3
Adjusted r^2	0	0.01	0.01	0.01	0.01	0.03	0.06	0.1	0.14	0.1

Scale (kb)

b Male recombination rate

	2	4	8	16	32	64	128	256	512	1024
Male DNA methylation	17.3	23	16	13	3.5	1.6	1.1	0.7	0.4	0.1
Female DNA methylation	49.7	60.7	59.7	33.4	13.1	1.7	0.1	0.4	0.3	0
H3K4me3	Inf	Inf	Inf	170.8	81.6	28.1	7	3.1	0.9	0.1
CpG islands	4.7	41.2	55	37.4	20.4	6.1	1.5	0.7	0.3	0.2
CpG-content	14	3.4	1.1	0.1	0.1	1.6	4.7	8.9	4.5	1
GC-content	10.2	24.7	78.5	109.5	100.1	121	107.6	61.4	20.9	2
Adjusted r^2	0	0.01	0.01	0.03	0.04	0.08	0.16	0.23	0.3	0.15

Scale (kb)

Significance level (p-value)

- <0.00001
- 0.0001-0.00001
- 0.001-0.0001
- 0.01-0.001
- 1
- 0.01-0.001
- 0.001-0.0001
- 0.0001-0.00001
- <0.00001

c Rate difference (female-male)

	2	4	8	16	32	64	128	256	512	1024
Male DNA methylation	0.9	0.8	0.2	0.3	0	0.1	0.1	0.1	0.6	0.5
Female DNA methylation	2.3	4.1	7.5	6.7	1.8	0.1	0.2	0.1	0.8	0.3
H3K4me3	8	9.4	9.6	8.9	1	0.2	0.1	0.7	0.5	0.2
CpG islands	3.2	0.3	1.2	1.6	0.1	3.1	0.3	0.5	0.5	0.6
CpG-content	0.8	0.1	1.3	0.3	0.3	2.2	1.7	5.3	2.1	0.2
GC-content	13.8	30.4	56	50.2	31.9	24.2	16.3	13	7	0.4
Adjusted r^2	0	0	0	0	0.01	0.01	0.02	0.04	0.08	0.04

Scale (kb)

Supplementary Figure 17. Epigenetic and genomic features associated with female and male recombination rates. Wavelet-based linear model of scale-specific correlates to the **(a)** female recombination rate, **(b)** male recombination rate and **(c)** recombination rate difference between female and male. Tables show the marginal significance ($-\log_{10}$ p-value two-sided t-test) for the linear model analyses of wavelet detail coefficients. Color indicates the direction of the relationship (red=positive; blue=negative) with intensity proportional to significance.

Supplementary References

1 Campbell, C. L., Furlotte, N. A., Eriksson, N., Hinds, D. & Auton, A. Escape from crossover interference increases with maternal age. *Nat Commun* **6**, 6260, doi:10.1038/ncomms7260 (2015).

2 Abecasis, G. R., Cherny, S. S., Cookson, W. O. & Cardon, L. R. Merlin--rapid analysis of dense genetic maps using sparse gene flow trees. *Nat Genet* **30**, 97-101, doi:10.1038/ng786 (2002).

3 Bleazard, T., Ju, Y. S., Sung, J. & Seo, J. S. Fine-scale mapping of meiotic recombination in Asians. *BMC Genet* **14**, 19, doi:10.1186/1471-2156-14-19 (2013).

4 Hinch, A. G. *et al.* The landscape of recombination in African Americans. *Nature* **476**, 170-175, doi:10.1038/nature10336 (2011).

5 Kong, A. *et al.* Common and low-frequency variants associated with genome-wide recombination rate. *Nat Genet* **46**, 11-16, doi:10.1038/ng.2833 (2014).

6 Fledel-Alon, A. *et al.* Variation in human recombination rates and its genetic determinants. *PLoS One* **6**, e20321, doi:10.1371/journal.pone.0020321 (2011).

7 Martin, H. C. *et al.* Multicohort analysis of the maternal age effect on recombination. *Nat Commun* **6**, 7846, doi:10.1038/ncomms8846 (2015).

8 Hussin, J., Roy-Gagnon, M. H., Gendron, R., Andelfinger, G. & Awadalla, P. Age-dependent recombination rates in human pedigrees. *PLoS Genet* **7**, e1002251, doi:10.1371/journal.pgen.1002251 (2011).

9 Kong, A. *et al.* Fine-scale recombination rate differences between sexes, populations and individuals. *Nature* **467**, 1099-1103, doi:10.1038/nature09525 (2010).

10 Coop, G., Wen, X., Ober, C., Pritchard, J. K. & Przeworski, M. High-resolution mapping of crossovers reveals extensive variation in fine-scale recombination patterns among humans. *Science* **319**, 1395-1398, doi:10.1126/science.1151851 (2008).

11 Kong, A. *et al.* Detection of sharing by descent, long-range phasing and haplotype imputation. *Nat Genet* **40**, 1068-1075, doi:10.1038/ng.216 (2008).

12 O'Connell, J. *et al.* A general approach for haplotype phasing across the full spectrum of relatedness. *PLoS Genet* **10**, e1004234, doi:10.1371/journal.pgen.1004234 (2014).
